# Age-specific rate of severe and critical SARS-CoV-2 infections estimated with multi-country seroprevalence studies

**DOI:** 10.1186/s12879-022-07262-0

**Published:** 2022-03-29

**Authors:** Daniel Herrera-Esposito, Gustavo de los Campos

**Affiliations:** 1 Laboratorio de Neurociencias, Facultad de Ciencias, Universidad de la República, Montevideo, Uruguay; 2 Centro Interdisciplinario de Ciencia de Datos y Aprendizaje Automático, Universidad de la República, Montevideo, Uruguay; 3 Department of Epidemiology and Biostatistics, Michigan State University, East Lansing, MI USA; 4 Department of Statistics and Probability, Michigan State University, East Lansing, MI USA; 5 Institute for Quantitative Health Science and Engineering, East Lansing, MI USA

**Keywords:** SARS-CoV-2, COVID-19, Severity, Critical disease, Meta-analysis, Serology

## Abstract

**Background:**

Knowing the age-specific rates at which individuals infected with SARS-CoV-2 develop severe and critical disease is essential for designing public policy, for infectious disease modeling, and for individual risk evaluation.

**Methods:**

In this study, we present the first estimates of these rates using multi-country serology studies, and public data on hospital admissions and mortality from early to mid-2020. We combine these under a Bayesian framework that accounts for the high heterogeneity between data sources and their respective uncertainties. We also validate our results using an indirect method based on infection fatality rates and hospital mortality data.

**Results:**

Our results show that the risk of severe and critical disease increases exponentially with age, but much less steeply than the risk of fatal illness. We also show that our results are consistent across several robustness checks.

**Conclusion:**

A complete evaluation of the risks of SARS-CoV-2 for health must take non-fatal disease outcomes into account, particularly in young populations where they can be 2 orders of magnitude more frequent than deaths.

**Supplementary Information:**

The online version contains supplementary material available at 10.1186/s12879-022-07262-0.

## Background

The SARS-CoV-2 pandemic had impacts of historic proportion in both public health and society. Remarkably, there is considerable uncertainty regarding the full spectrum of health effects of SARS-CoV-2 infection. On the one hand, some of the effects of SARS-CoV-2 are relatively well understood, such as the infection fatality rate (IFR) and its dependence on age. Three different meta-analyses now exist that estimate the age-stratified IFR of SARS-CoV-2 using multi-country seroprevalence studies [1–3]. These studies document an exponential increase of the IFR with age and show considerable agreement on their estimated IFRs by age-stratum. On the other hand, the rate of less extreme infection outcomes, and their dependence on age, remains uncertain despite being similarly important for public health. Examples of this are the rate of severe infections (Infection-severe rate, ISR), which we define as infections resulting in hospitalization or out-of-hospital death, and the rate of critical infections (Infection-critical rate, ICR), which we define as infections resulting in admission to intensive care unit (ICU) or out-of-ICU death.

But despite their relevance to analyzing the development of the pandemic and for future planning, estimates of ISR and ICR for these ages using multi-country data are still missing from the literature (see estimates of the infection-hospitalization rate, for France [4, 5], for Denmark [6], for Indiana, USA [7], for Connecticut, USA [8], for Qatar [9], and a model-based analysis with early non-serological pandemic data [10]). To fill this gap, we present a meta-analysis of the age-stratified rates of severe and critical disease of SARS-CoV-2 across several locations, combining seroprevalence studies from early to mid 2020 with public data on the numbers of age-stratified hospitalizations, ICU admissions, and deaths.

## Results

We analyzed locations with seroprevalence studies that were either listed in the meta-analysis of Levin et al. [1], to which we refer for further details, or the studies providing their own age-stratified rates of infection-hospitalization rate. We included in the analysis 15 locations with serosurveys (11 using representative samples and 4 using convenience samples), and 2 locations with comprehensive testing and contact tracing (see methods section M1 for a description of this classification of locations). Together, these locations represent 5% of the world’s population. Furthermore, to account for out-of hospital and out-of ICU deaths, which are common among the elderly, we computed the number of severe cases as the number of hospitalizations plus out-of-hospital deaths, and the number of critical cases as the number of ICU admissions plus out-of-ICU deaths.

**Fig. 1:**
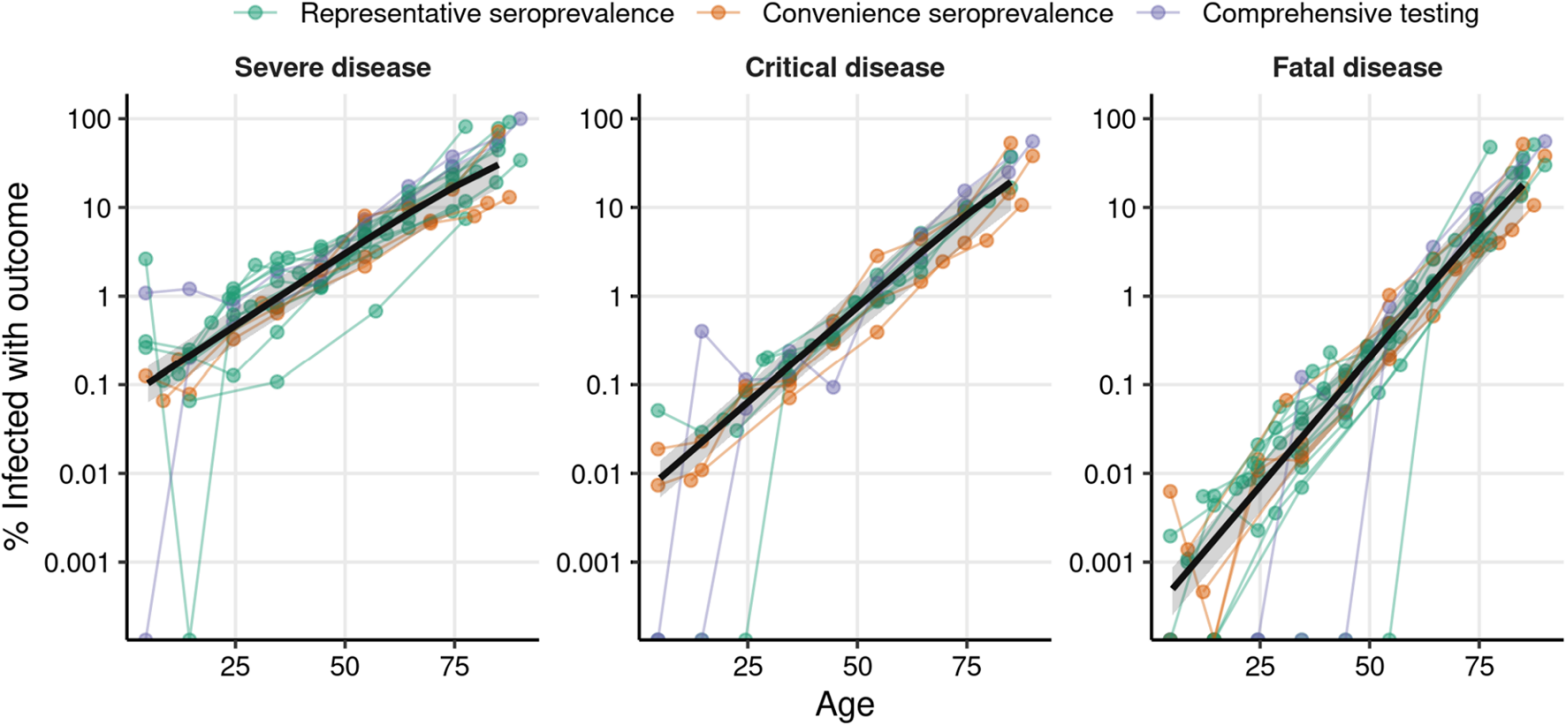
Rates of severe and critical SARS-Cov-2 outcomes (ISR and ICR, respectively) and death rates (IFR) estimated with seroprevalence data from 2020. The colored points show the proportion of individuals infected with SARS-CoV-2 that develop severe disease (left), critical disease (center), or fatal disease (right) (in logarithmic scale) for each location and age-stratum used in our analysis. Color indicates whether the number of infections were obtained from a representative serosurvey, a convenience serosurvey, or from comprehensive testing corrected for under-ascertainment. Data points coming from a given location are joined by colored lines. The black line shows the outcome rate estimated using a hierarchical Bayesian logistic regression model, and the shaded regions show the 95% credibility intervals. We used 105 data points from 16 locations for the estimation of ISR, 78 data points from 11 locations for ICR, and 119 data points from 17 locations for IFR

The estimated probability of severe, critical, and fatal disease outcomes (ISR, ICR, and IFR, respectively) are shown for each age and location as colored points in Fig. 1, using a log-transformed vertical axis. As expected from the reports in previous analyses of IFR [1–3] and of the infection-hospitalization ratio [4], the three outcome ratios show an approximately exponential increase in risk with respect to age, which becomes a homogeneous linear effect on the log-scale. Thus, for each outcome rate (ISR, ICR, and IFR) we fitted Bayesian logistic regression models with a linear age effect on the logit scale (this effect becomes non-linear in the risk scale). We used logistic regression because it is a commonly used model for disease outcomes that approximate the log-linear rate-age patterns observed in our study. The logistic regression model had an intercept and age-slope shared across locations, plus location-specific random effects on both the slope and the intercept of the regression to account for the heterogeneity between locations. We also accounted for the uncertainty of the seroprevalence estimates (through the specification of the prior distribution), and the sampling (binomial) variability of the observed outcomes.

The logistic regression models fitted the data well for the three outcome rates (Fig. 1, although see the discussion of section S1 in Additional file 1 about a possible deviation from the trend for the youngest ages), and the patterns observed were similar across locations. Importantly, the slope of IFR with respect to age (0.133, 95% credibility interval: [0.123–0.143]) was higher than the slope of ICR (0.099, [0.089–0.108]) and of ISR (0.076, [0.067–0.083]), indicating that the risks of severe and critical disease are more evenly distributed across ages than the risk of death. The models also had different intercepts, of − 12.9 [− 13.6, − 12.3] for IFR, − 9.9 [− 10.3, − 9.4] for ICR and − 7.3 [− 7.7, − 6.8] for ISR, reflecting the difference of 1 order of magnitude between ICR and IFR for the youngest ages, and of 2 orders of magnitude between ISR and IFR. As an example, according to our estimates, people in the 20–25 years old range are on average 779 [467–1223] times less likely to die from COVID-19 than 70–75 year old people, 133 [83–202] times less likely to develop critical disease, and 38 [27–51] times less likely to develop severe disease.

Predicted risk levels by age (and the corresponding 95% credibility intervals) are shown in Table 1 (see Table S1 for finer age stratification, and Table S2 for the estimated model parameters in Additional file 1). Also, we verified that our estimates are robust to the correction for out-of-hospital and out-of-ICU deaths (Additional file 1: Figs. S1, S2), the ages used to fit the model (Additional file 1: Fig. S3), the method of estimating SARS-CoV-2 infections (Additional file 1: Fig. S4), the date of outcome data collection (Additional file 1: Figs. S5, S6), and the delay between the epidemic wave and the seroprevalence study (Additional file 1: Fig. S7, to control for seroreversion). More details about these comprehensive robustness analyses are provided in the Additional file 1.

**Table 1:** Estimated age-specific frequencies of severe disease (ISR), critical disease (ICR), and fatal disease (IFR) among infected individuals

Age	ISR % (CrI)	ICR % (CrI)	IFR % (CrI)
0–9	0.103 (0.063–0.162)^a^	0.0088 (0.0053–0.0139)^a^	0.00050 (0.00025–0.00087)^a^
10–19	0.22 (0.13–0.35)	0.024 (0.014–0.037)	0.0019 (0.0010–0.0033)
20–29	0.47 (0.28–0.74)	0.063 (0.038–0.10)	0.0072 (0.0037–0.0126)
30–39	0.99 (0.57–1.61)	0.17 (0.10–0.28)	0.027 (0.014–0.049)
40–49	2.1 (1.2–3.5)	0.46 (0.26–0.77)	0.10 (0.05–0.19)
50–59	4.4 (2.4–7.4)	1.2 (0.6–2.1)	0.40 (0.18–0.77)
60–69	8.9 (4.6–15.2)	3.3 (1.6–5.9)	1.5 (0.6–3.0)
70–79	17.1 (8.9–28.8)	8.3 (3.9–15.5)	5.5 (2.3–11.3)
80 +	30.3 (16.4–47.7)	19.4 (9.2–34.7)	18 (7.5–34.3)

The estimates are obtained from the fits to the serology data from 2020 shown in Fig. 1. Numbers in the parenthesis indicate 95% credibility intervals of the estimates, obtained by taking the 2.5% and 97.5% quantiles of the posterior probability of the bayesian fit

^a^Estimates for the youngest ages may be underestimated by the assumption of a logistic relation between age and severity, see section S1 in Additional file 1 for further discussion and complementary estimates

**Fig. 2:**
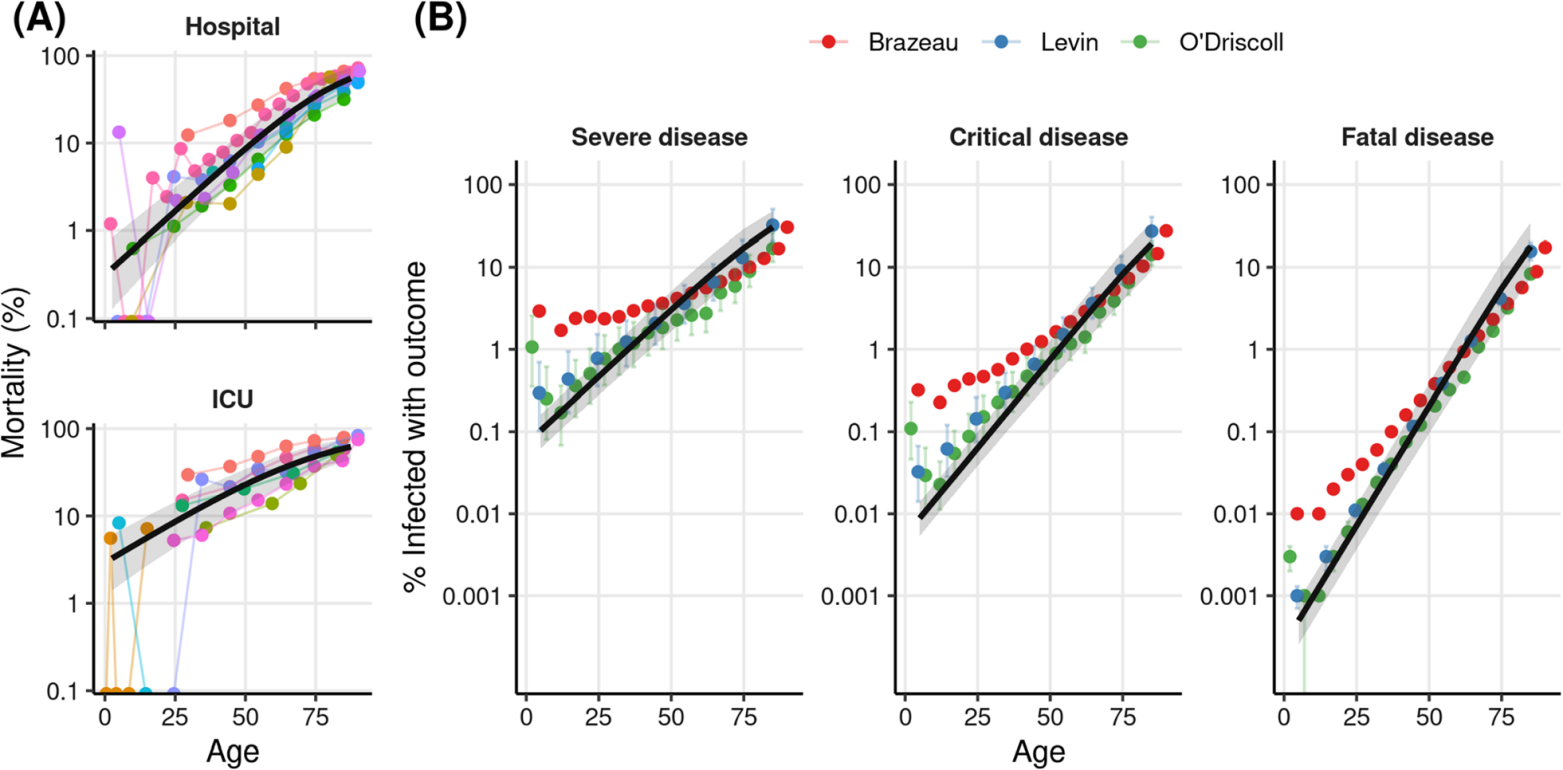
Rates of severe (ISR) and critical (ICR) obtained with an indirect method based on a ratio of ratios. **A** The colored points represent the reported mortality rates of hospitalized (top) and ICU SARS-Cov-2 patients, each study is reported in a different color. The black line shows the estimated outcome rates for each age obtained from our hierarchical Bayesian logistic regression, and the shaded regions show the 95% credibility intervals. 68 data points from 8 reports were used for hospital mortality, and 43 data points from 8 reports were included for ICU mortality. **B** The colored points show the estimated rates of severe (left) and critical (center) disease, obtained by dividing the age-stratified IFRs of the three relevant meta-analyses [1–3] by the corresponding values obtained in **A**. The points show the mean values of the posterior distribution, and bars show 95% credibility intervals (we omit these for Brazeau et al. since the credibility intervals around the mean estimates are not reported). The rightmost plot shows the IFRs reported by each of the studies. The black line and shaded region in each panel show the meta-analysis estimates we obtained with the direct method (Fig. 1)

Next, we validated our estimates by estimating the ISR and ICR of SARS-CoV-2 indirectly using a novel ratio-of-ratios approach. We start from the age-specific IFR reported in the three different meta-analyses [1–3], which were not used in the analysis of Fig. 1. Because the IFR is the expected ratio between deaths and infections, we can estimate the ISR as the ratio IFR/SFR, where SFR is the ratio between deaths and severe infections (severe fatality rate). We approximated the age-specific SFR by fitting a Bayesian logistic regression model to published data of COVID-19 hospital mortality (Fig. 2A, data sources listed in Table 4), which is the ratio between in-hospital deaths and hospitalizations. The approximation of SFR by hospital mortality assumes that all deaths occur in hospitals, which is expected to hold well for all but the oldest age bins (see Additional file 1: Section S2, Fig. S2). Then, we estimated the age-specific ISR by taking the ratio between the IFRs and the SFRs. We applied the same procedure to estimate ICR, using the ICU mortality of COVID-19 patients. The values of the parameters obtained by fitting the model to hospital and ICU mortality are shown in Additional file 1: Table S3, and the age-specific estimates are shown in Additional file 1: Table S4.

We note that we used the same hospital and ICU mortality data for this analysis and for correcting for out-of-hospital and out-of-ICU deaths in Fig. 1. Although this means that the two analyses share some data in common, the results of the regression of Fig. 1 are similar when performed on the uncorrected data (Additional file 1: Figs. S1, S2), supporting the use of this validation method.

The estimates from the indirect method are shown by the colored points in Fig. 2B. To aid comparison, we show in black the lines obtained from the fit to serology data of Fig. 1. Firstly, we see that our IFR estimates and the IFR estimates from the three meta-analyses are very similar (Fig. 2B, right). Second, we note that the estimates of ISR and ICR obtained from seroprevalence and disease outcome data with the direct method (Fig. 1) are in close agreement with the estimates obtained with the indirect method (with the largest differences being with Brazeau et al. [3] for the younger ages; however, this study also reports estimates different than those reported in the other two studies).

## Conclusions

In conclusion, we present estimates of the rates of severe and critical SARS-CoV-2 infections during the first half of 2020. These are the first estimates based on multi-country seroprevalence data, which we combine in a rigorous way using Bayesian methods, to account for the uncertainty of each study as well as temporal and geographical heterogeneity. We find that while young and middle-aged individuals had low rates of fatal infection, they had much higher rates of severe and critical infection, emphasizing the need to consider these disease outcomes in these populations. The estimates presented here are an important reference of the health impacts of COVID-19 during 2020, as well as an important baseline over which to build more updated estimates, by combining them with estimates of the relative change in risk across locations and time.

## Methods

The data sets included in this study come from locations where age-stratified seroprevalence studies have been performed (see M1), plus locations with age-stratified prevalence coming from exhaustive contract tracing (see M2). For each of these locations, we searched for age-stratified data on Hospitalizations and ICU admissions (see M3). We used these two sources of data to estimate the age-stratified rates of severe (ISR) and critical (ICR) SARS-CoV-2 infections for each of the locations. We used this data to fit Bayesian random-effects logistic regression models for each of the outcomes (see M4). We also searched for studies reporting age-stratified mortality for COVID-19 patients admitted to the hospital or to the ICU (see M5). We used this data to fit a Bayesian random-effects logistic regression model to obtain the age-specific hospital and ICU mortality for COVID-19. This regression was then combined with estimates of age-specific IFR extracted from the literature, to estimate the ISR and ICR through a ratio-of-ratios method (see M6). The regression of hospital and ICU mortality was also used to correct the hospital and ICU data described in M3 for out-of-hospital and out-of-ICU deaths (see M7). All data and code are available online (see M8).

### (M1) Data from seroprevalence studies

We used a curated list of seroprevalence studies released prior to 18 September 2020 that is presented in Levin et al. [1]—a systematic review and a meta-analysis. The list is restricted to developed countries; we refer the reader to Levin et al. [1] for an exhaustive list of other existing studies, and the criteria used for excluding seroprevalence studies from their final analysis. The locations used by Levin et al. [1] can be divided into three groups: those with representative seroprevalence studies, those with convenience seroprevalence studies, and those with comprehensive testing and tracing. Representative seroprevalence studies refer to those in which the population included in the serosurvey aims to be a representative sample of the population. Convenience seroprevalence studies are those that perform the serosurvey over samples that are conveniently available but not necessarily representative of the population, like blood donor samples. Locations with comprehensive testing and tracing are defined by Levin et al. [1] as locations that up to the date of interest, had over 300 tests performed for each detected case (in these locations, we corrected for under-ascertainment following Levin et al. [1]).

We then searched for age-stratified hospitalization and ICU data to match the representative seroprevalence studies listed in Additional file 1: Appendix Tables I1 and I3, and the convenience seroprevalence studies listed in Appendix Table I2 from Levin et al. [1]. From the 11 representative seroprevalence studies included in those lists, we were able to find age-stratified hospitalization or ICU data for 7 locations (England; France; Ireland; Netherlands; Spain; Atlanta, USA; Geneva, Switzerland) and we failed to find such data for the 4 remaining locations (Italy; Portugal; Indiana, USA; Salt Lake City, USA). From the 4 convenience seroprevalence studies listed, we were able to find hospitalization or ICU data for all three locations (Ontario, Canada; Sweden; Belgium; New York, USA).

In addition to the seroprevalence studies used in Levin et al. [1], we included the seroprevalence study carried out in Iceland up to April 4 2020, which reports the results of a representative sample of the population [46], and the representative seroprevalence studies from Indiana, Connecticut and Denmark, which were used to estimate the ISRs in the existing literature [6–8].

Furthermore, in three cases we also changed the use of some seroprevalence studies with respect to Levin et al. to match them to the available hospitalization and ICU data. The first case is the seroprevalence study from France, which offers data by region. We were only able to find the age-stratified hospitalization data for the region of Île-de-France; therefore, we only used seroprevalence data from this region. The second case is the New York seroprevalence study, where we could only find hospitalization data for New York City but not for New York State; thus, we only used the seroprevalence for New York City. For Ontario, Canada, we could only find age-stratified hospitalization and ICU data up to July 31 2020, and so we used the seroprevalence report for this date, which is different from the report date used by Levin et al. [1]. Table 2 summarizes the final list of seroprevalence studies included in our analysis.

**Table 2:** List of sources for the seroprevalence data of each location

Location	End date	Source
England (< 17 years)	03/07/2020	[47]
England	13/07/2020	[48]
Ile-de-France	23/06/2020	[49]
Ireland	16/07/2020	[50]
Netherlands	11/05/2020	[51]
Spain	11/05/2020	[52]
Atlanta, USA	03/05/2020	[53]
New York City, USA	28/04/2020	[54]
Ontario, Canda	31/07/2020	[55]
Sweden	24/05/2020	[56]
Iceland	04/04/2020	[46]
Geneva, Switzerland	06/05/2020	[57]
Belgium	26/04/2020	[58]
Connecticut, USA	29/07/2020	[59]
Indiana, USA	29/04/2020	[7]
Denmark	16/12/2020	[6]

The final date of the data collection period is shown in the center column

#### Matching age-bins

The age-bins reported by each of the studies did not always match the age-bins in the corresponding hospitalization and ICU reports. Therefore, in some cases we extrapolated or interpolated the seroprevalence estimates obtained for a given age-bin into a different age-bin. For example, for New York City, seroprevalence was reported for the 18–34 years old age range, but hospitalization data was reported for the 18–44 year old age range. Therefore, to make use of this hospitalization data, we assumed that the proportion of seropositive individuals in the 18–44 years old range is the same as the proportion for the 18–34 year old age range. All such decisions were contrasted with other available data, and agreed upon by the two authors. Furthermore, these assumptions are all documented in the publicly available analysis code.

#### Correcting for test characteristics

The positive rate of a test depends on disease prevalence and on the test characteristics. Most of the seroprevalence estimates used were already corrected for test characteristics. For the results that were not corrected for test characteristics, we used the Gladen-Rogan formula (Rogan and Gladen 1978) to adjust the estimates as follows:

$$prevalence=\frac{test positive rate+specificity-1}{sensitivity+specificity-1}.$$

### (M2) Countries with comprehensive tracing included in the analysis

Following [1], we also included in our analysis two countries (Republic of Korea and New Zealand) with comprehensive tracing programs where the number of infections detected through testing are thought to approximate the total number of infections accurately. As in the original Levin et al. study, we corrected the prevalence estimates for these countries using the age-specific ratio between the number of infections estimated through seroprevalence and the number of positive tests in Iceland (Gudbjartsson et al. 2020).

### (M3) Hospitalizations, ICU admissions, and deaths data

We obtained the age-stratified hospitalizations, ICU, and death data in relevant government websites of the locations, using google search, and looking for relevant region-wide studies. We selected the data reports that were closest to the end of the serosurvey date. The list of data sources and the end dates for their cumulative outcome numbers are shown in Table 3.

**Table 3:** List of sources for the hospitalization, ICU, and death data for each location

Location	Date of outcome data	Final date of serosurvey	Outcome data source
Spain	11/05/2020	11/05/2020	https://cnecovid.isciii.es/covid19/#documentaci%C3%B3n-y-datos
Ireland	16/07/2020	16/07/2020	https://www.hpsc.ie/a-z/respiratory/coronavirus/novelcoronavirus/casesinireland/epidemiologyofcovid-19inireland/july2020/
Sweden (ICU)	24/05/2020	24/05/2020	https://portal.icuregswe.org/siri/report/corona.alderkon?filter=b213d908-6dcf-d4be-c121-8eeda3a9578a
Sweden (Deaths)	24/05/2020	24/05/2020	https://www.socialstyrelsen.se/statistik-och-data/statistik/statistik-om-covid-19/statistik-over-antal-avlidna-i-covid-19/
Ile-de-France, France	26/05/2020	23/06/2020	https://www.santepubliquefrance.fr/regions/ile-de-france/documents/bulletin-regional/2020/covid-19-point-epidemiologique-en-ile-de-france-du-28-mai-2020
England (ICU)	03/07/2020	13/07/2020	ICNARC report on COVID-19 in critical care 03 July 2020
England (Hospital)	13/07/2020	13/07/2020 for 18 + years, 03/07/2020 for 0–17 years	https://coronavirus.data.gov.uk/details/download
England (Deaths-1)	13/07/2020	13/07/2020 for 18 + years, 03/07/2020 for 0–17 years	https://www.ons.gov.uk/peoplepopulationandcommunity/birthsdeathsandmarriages/deaths/bulletins/deathsregisteredweeklyinenglandandwalesprovisional/weekending18june2021
England (Deaths-2)	29/07/2020	13/07/2020 for 18 + years, 03/07/2020 for 0–17 years	Levin et al. [1]
Netherlands (Hospital and deaths)	11/05/2020	11/05/2020	https://data.rivm.nl/geonetwork/srv/dut/catalog.search#/metadata/2c4357c8-76e4-4662-9574-1deb8a73f724?tab=general
Netherlands (Deaths-2)	11/05/2020	11/05/2020	https://www.rivm.nl/documenten/epidemiologische-situatie-covid-19-in-nederland-11-mei-2020
Netherlands (deaths under 50)	11/05/2020	11/05/2020	https://www.rivm.nl/documenten/epidemiologische-situatie-covid-19-in-nederland-11-mei-2020
New York City, USA	28/04/2020	28/04/2020	https://www1.nyc.gov/site/doh/covid/covid-19-data-archive.page
Ontario, Canada	31/07/2020	31/07/2020	https://covid-19.ontario.ca/covid-19-epidemiologic-summaries-public-health-ontario
Toronto, Canada	31/07/2020	30/07/2020	https://public.tableau.com/app/profile/tphseu/viz/EpidemiologicalSummaryofCOVID-19Cases/EpiSummary
New Zealand	13/01/2021	13/01/2021	https://www.health.govt.nz/our-work/diseases-and-conditions/covid-19-novel-coronavirus/covid-19-data-and-statistics/covid-19-case-demographics
New Zealand (out of hospital and ICU deaths)	13/01/2021	13/01/2021	By mail at data-enquiries@health.govt.nz
Atlanta, USA (unstratified counts)	08/05/2020	03/05/2020	https://www.fultoncountyga.gov/covid-19/epidemiology-reports
Atlanta, USA (age distributions)	31/05/2020	03/05/2020	[60]
Geneva, Switzerland	10/05/2020	06/05/2020	https://www.covid19.admin.ch/en/weekly-report/hosp?geoView=table
Belgium (Hospital and ICU)	08/05/2020	26/04/2020	https://covid-19.sciensano.be/fr/covid-19-situation-epidemiologique
Belgium (deaths)	09/05/2020	26/04/2020	[61]
Iceland	16/06/2020	04/04/2020	Personal communication with the authors of Eythorsson et al. [62]
Republic of Korea	30/04/2020	30/04/2020	[63]
Connecticut, USA (Hospital)	01/06/2020	29/07/2020	[8]
Connecticut, USA (Deaths)	01/06/2020	29/07/2020	https://data.ct.gov/Health-and-Human-Services/COVID-19-Cases-and-Deaths-by-Age-Group/ypz6-8qyf/data
Indiana, USA (ICU, Deaths)	30/04/2020	29/04/2020	[7]
Indiana, USA (Hospital)	14/05/2020	29/04/2020	https://www.regenstrief.org/covid-dashboard/
Indiana, USA (Deaths-2)	14/05/2020	29/04/2020	https://www.in.gov/mph/
Denmark	12/12/2020	16/12/2020	[6]

The date up to which the cumulative numbers for these outcomes were reported are shown in the second column

Also, as described above for the serology data, in some cases we interpolated or extrapolated some data for these disease outcomes, or we combined different data sources with incomplete data (e.g., age-distribution of an outcome from one source, with the total count of the outcome from another source) to obtain the data for these outcomes with the appropriate age bins. For example, for Belgium, we were only able to find the unstratified number of cumulative ICU admissions at the desired date of May 8th, 2020, but we were able to find the age distribution for cumulative ICU admissions up to June 14th, 2020. Therefore, we distributed the cumulative ICU admissions of May 8th across age strata, following the distribution from June 14th. As mentioned above, all these decisions were agreed upon by the authors, and they are thoroughly documented in the publicly available analysis code.

### (M4) Estimation of outcome probabilities with serology and outcome data

We fitted Bayesian logistic regression models to the serology and outcome data. We describe the model for severe SARS-CoV-2 outcome; the same model was fitted to severe, critical, and fatal disease outcomes.

Let $\{{y}_{la},{x}_{la}\}$ represent the number of severe SARS-Cov-2 infections ${(y}_{la})$ experienced among ${x}_{la}$ individuals infected with SARS-CoV-2, at the location *l* (*l* = 1, …, L), for the age stratum *a* (*a* = 1, …, A_*l*_) of the *l*^*th*^ location. The ISR for this location-stratum is defined as ${\theta }_{R,la}=E[\frac{{y}_{la}}{{x}_{la}}]$.

#### Bayesian likelihood

The probability of ${y}_{la}$ given ${\theta }_{R,la}$ and the number of infections${(x}_{la})$, is given by the Binomial likelihood$p({y}_{la}) \propto {\theta }_{R,la}^{{y}_{la}}(1-{\theta }_{R,la}{)}^{{x}_{la}-{y}_{la}}$. Assuming conditional independence across locations and strata, and taking$y=({y}_{11},{y}_{12}, ...,{y}_{L{A}_{L}})$, and ${\theta }_{R}=({\theta }_{R,11},{\theta }_{R,12}, ...,{\theta }_{R,L{A}_{L}})$ we have $p({y|\theta }_{R}) \propto {\prod }_{l=1}^{l=L} {\prod }_{a=1}^{a={A}_{l }}{\theta }_{R,la}^{{y}_{la}}(1-{\theta }_{R,la}{)}^{{x}_{la}-{y}_{la}}$

#### Modeling the number of SARS-Cov-2 cases from seroprevalence data

The selected seroprevalence studies provide age-stratified estimates (and SE) of disease prevalence. Rather than assuming that prevalence was known with complete certainty, we used the reported point estimates and SE to specify a Beta prior for prevalence for each location. Specifically, we used the reported prevalence and its SE to estimate (through first and second moments matching) the shape parameters of the Beta distribution used for each location. Then, prevalence was modeled as ${\theta }_{P,la}\sim Beta({\alpha }_{1,la},{\alpha }_{2,la})$, where ${\alpha }_{1,la}$ and ${\alpha }_{2,la}$ are location-age-stratum specific shape parameters. Then, the number of cases was defined as ${x}_{la}={N}_{la}\times {\theta }_{P,la}$, where ${N}_{la}$ is the size of the population at location *l* and age stratum *a*.

#### Modeling severity rates using random-effects logistic regression

Infection-severity rates were modeled using a logit of the form.

$$log\left(\frac{{\theta }_{R,la}}{1-{\theta }_{R,la}}\right)=\left[\mu +{u}_{l}\right]+ \left[\beta +{b}_{l}\right]\times ag{e}_{la}.$$

Therefore, $${\theta }_{R,la}=\frac{{e}^{{\eta }_{la}}}{1+{e}^{{\eta }_{la}}}$$ where $${\eta }_{la}=[\mu +{u}_{l}] + [\beta +{b}_{l}]\times ag{e}_{la}$$.

Above, $\mu$ and $\beta$ are the average intercept and slopes across locations, and ${u}_{l}$ and ${b}_{l}$ are location-specific random effects on the intercept and the slope, respectively, with prior distribution $p(u,b|{\sigma }_{\mu }^{2},{\sigma }_{b}^{2})={\Pi }_{l=1}^{L}N({u}_{l}|0,{\sigma }_{u}^{2})N({b}_{l}|0,{\sigma }_{b}^{2})$. The shared intercept ($\mu$) and regression coefficient ($\beta$) were assigned flat priors, and the standard deviations for the random effects, ${\sigma }_{u}$ and ${\sigma }_{b}$ were assigned gamma priors with shape and rate parameters equal to 4.

For each age stratum *a* at location *l*, the value of $ag{e}_{la}$ used for fitting corresponded to the median age of the stratum. For age strata with an open upper bound (e.g. 70 + age), we used 90 years as the upper bound of the stratum.

The posterior distribution of the model described above does not have a closed form; therefore, we used Monte Carlo Markov Chain (MCMC) methods to generate samples from the posterior distribution for all the model unknowns $\{\mu ,\beta ,x,u,b,{\sigma }_{\mu }^{2},{\sigma }_{b}^{2}\}$. We used 4 chains with 2500 iterations each. A script that implements the above model in Stan [64] is available in the online code.

#### Prediction of outcome rates

We used the samples of the posterior distribution to generate posterior samples for the infection severity rates for specific ages using the inverse-logit function: ${\theta }_{R}(age{)}_{s}=\frac{{e}^{{\mu }_{s}+age\times {\beta }_{s}}}{1+{e}^{{\mu }_{s}+age\times {\beta }_{s}}}$, where $s$ is an index for the sample from the posterior distribution. We then used these samples to estimate the posterior means and posterior credibility regions reported in Figs. 1 and 2. We report the severity rates for age intervals by estimating the rate of the mean age of the interval.

The predicted outcome rates obtained from the model fit are shown in Additional file 1: Table S1, and the mean and credible intervals for the main model parameters are shown in Additional file 1: Table S2.

### (M5) Hospital and ICU mortality data

Our robustness analysis was based on an indirect estimator (a ratio-of-ratios) of ISR and ICR. To derive this estimator, we used mortality data from hospitalized and ICU SARS-Cov-2 patients. We searched in the literature for reports on age-stratified mortality of patients admitted to the hospital or the ICU with a COVID-19 diagnosis. We also used the data sources from Table 3 that provided mortality numbers for hospitalized or ICU patients. We identified 8 ICU mortality reports and 8 mortality hospital reports with age-stratified data, which were either published studies in the literature or public reports from official organisations. The reports used are listed in Table 4.

**Table 4:** List of sources for mortality among COVID-19 patients in the hospital or in critical care

Location	Patient type	Date	Source
England	ICU	03/07/2020	ICNARC report on COVID-19 in critical care 03 July 2020
New York City, USA	ICU	28/04/2020	[65]
France, Belgium, Switzerland	ICU	04/05/2020	[66]
Sweden	ICU	01/09/2020	https://portal.icuregswe.org/siri/report/corona.alderkon?filter=b213d908-6dcf-d4be-c121-8eeda3a9578a
Brazil	ICU	15/08/2020	[67]
Florida, USA	ICU	18/05/2020	[68]
Intercontinental	ICU	23/04/2020	[69]
Brazil	ICU	31/05/2020	[70]
New York City, USA	Hospital	04/04/2020	[71]
Germany	Hospital	19/04/2020	[72]
France	Hospital	13/05/2020	[73]
United Kingdom	Hospital	19/04/2020	[74]
Spain	Hospital	17/04/2020	[75]
Chile	Hospital	04/06/2020	[76]
Brazil	Hospital	15/08/2020	[67]
Netherlands	Hospital	11/05/2020	https://data.rivm.nl/geonetwork/srv/dut/catalog.search#/metadata/2c4357c8-76e4-4662-9574-1deb8a73f724?tab=general

The end date of each study is shown in the third column

### (M6) Indirect estimation of ISR and ICR using IFR and hospital mortality data

To validate the estimates obtained with the data and methods described above, we used an alternative source of data and a different estimation method to obtain age-specific ISR and ICR. Specifically, we combined age-specific reports of IFRs from the literature with the hospital and ICU mortality data listed in Table 4 to obtain the ISR and ICR using a ratio-of-ratios method, as explained below.

Let $IF{R}_{sa}$ be the expected ratio between deaths and infections estimated in a study *s* (s = 1, …, S) for age stratum *a* (*a* = 1, …, *A*_*s*_) and let $SF{R}_{a}$ be the expected ratio between deaths and severe COVID-19 cases for age stratum *a* (*a* = 1, …, *A*_*s*_). Then, we have that the estimated ISR for age stratum *a* estimated from study *s* is $IS{R}_{sa}=\frac{IF{R}_{sa}}{SF{R}_{a}}$. Thus, by estimating the values of $SFR$ for different ages, we can use age-specific IFR values reported in the literature to obtain estimates of age-specific ISR.

To approximate the age-specific SFR, we fitted a Bayesian logistic regression to age-stratified hospital death for COVID-19 patients. Let $\{{d}_{la},{h}_{la}\}$ represent the number of deaths ${(d}_{la})$ among ${h}_{la}$ individuals hospitalized with COVID-19 for the age stratum *a* (a = 1, …, A_*l*_) in location *l*. The hospital mortality for this location-stratum is defined as ${\theta }_{HM,la}=E\left[\frac{{d}_{la}}{{h}_{la}}\right]$.

To estimate ${\theta }_{HM}(age)$, we used Bayesian random-effects logistic regressions, like the one described in section M4, to the hospital death data. The only difference with the procedure in M4 is that, in this case, the denominators ${h}_{la}$ were known, and thus we directly used these fixed ${h}_{la}$ values (unlike the ${x}_{la}$ from M4, for which a distribution over possible values was obtained using seroprevalence estimates).

We use ${\theta }_{HM}(a)$ as our estimate of $SF{R}_{a}$. These two quantities are equal if we assume that all deaths occur in the hospital (note that our definition of severe case, stated in the main text, is a case that results in either hospital admission or out-of-hospital death). As discussed in section S3 and shown in Additional file 1: Figs. S1, S2, out-of-hospital deaths make only a very small fraction of severe cases for all but the oldest age-strata. Also, we find that out-of-hospital deaths make up a minority of the deaths for all but the oldest ages (analysis not shown).

Then, to account for the uncertainty of the $IF{R}_{sa}$ estimates in our estimations, we fitted a Beta distribution to the mean and credible interval of each $IF{R}_{sa}$ through moment matching, to obtain $IF{R}_{sa}\sim Beta({\alpha }_{1,sa},{\alpha }_{2,sa})$ (for Brazeau et. al. (2020) we only used the point estimates since credible intervals on the mean estimates are not reported).

Finally, we estimated $IS{R}_{sa}=\frac{IF{R}_{sa}}{SF{R}_{a}}$ by generating samples from the posterior distribution of each $SF{R}_{a}$ (obtained from the Bayesian logistic regression model) and from the Beta distribution fitted for each $IF{R}_{sa}$. In total, we generated 50.000 samples of this ratio for each $IS{R}_{sa}$.

The same procedure was applied to estimate the $IC{R}_{sa}$, by fitting the model to ICU death data. The estimated hospital and ICU mortality rates obtained from these models are shown in Additional file 1: Table S3, and the parameters obtained from fitting the model are shown in Additional file 1: Table S4.

### (M7) Correction for out-of-hospital and out-of-ICU deaths

Some COVID-19 deaths occur outside of the ICU, or outside of the hospital. This happens when the patient prognosis is poor, such as in elderly and frail patients, and it may be accentuated when health systems are operating at high occupancy. This phenomenon is particularly notable in our data for some locations and ages, where the number of reported deaths is larger than the number of reported ICU admissions (in some cases by more than one order of magnitude).

Our definitions of severe and critical COVID-19 outcomes include these out-of-hospital and out-of-ICU deaths, besides hospitalizations and ICU admissions. Therefore, we obtained the number of severe cases by adding to hospitalizations the number of out-of-hospital deaths. Likewise, we obtained the number of critical cases by adding to the number of ICU patients the number of out-of-ICU deaths. For some locations, we could obtain data on the out-of-hospital and out-of-ICU deaths, but for other locations this data was absent, and so we estimated it using the death data.

Let ${y}_{la}$ be the cumulative number of hospitalizations for a location *l* and age stratum *a*, for which no out-of-hospital death data is available. Also, let ${{m}_{la}^{tot}}$ be the total number of deaths reported for this location and age stratum. First, we obtained the expected number of in-hospital-deaths, ${m}_{la}^{h}$, by combining the number of hospitalizations with the expected hospital mortality for this age, ${\theta }_{HM}(a)$ (fitted as described in section M6), ${m}_{la}^{h}={{y}_{la}\times {\theta }_{HM}(a)}$. Then, we obtain the expected number of out-of-hospital ${m}_{la}^{ooh}$ deaths by subtracting from the total number of deaths, ${m}_{la}^{ooh}={m}_{la}^{tot}-{m}_{la}^{h}$ (setting ${m}_{la}^{ooh}$ to 0 if the result is negative).

The same procedure is performed for the ICU data to obtain the number of critical cases.

See sections Additional file 1: Supplementary S3 and Figs. S1, S2 for an analysis showing the effect of this correction method on the data, and the robustness of the results to the removal of this correction.

## Discussion

In this work, we present the first estimates of ISR and ICR of SARS-CoV-2 obtained through a meta-analysis of serology studies from early to mid 2020. Our estimates show that, like the IFR, the ISR and ICR increase exponentially with age; however, the rate of increase in the risk of severe and critical disease outcomes with age is smaller than the rate of increase in lethality, which is in agreement with previous studies [5–11]. However, previous studies show considerable variability, probably due to the uncertainty in serology estimates, differences in local reporting protocols, and geographical variability in the impacts of COVID-19. Thus, this analysis presents the most up to date estimation and comparison of these rates, summarizing the best available evidence of several locations with a Bayesian approach. Our simple Bayesian regression analysis that takes into account several sources of uncertainty and this novel ratio-of-ratio methods constitute two complementary methods that may aid future work on estimating these parameters under the changing nature of the pandemic. For example, these methods can be extended so as to estimate the outcome rates for the new variants of SARS-CoV-2.

Furthermore, we provide extensive validation of our estimates (see Additional file 1). First, we performed several robustness analyses, controlling for various potential sources of bias in estimates. In Additional file 1: Fig. S3 we show that despite adverse outcomes concentrating in the older ages (giving them more statistical weight), the estimates for younger ages are robust to excluding older ages from the regression. In Additional file 1: Fig. S4 we show that our estimates are also robust to excluding the locations where prevalence was estimated from non-representative samples, which have increased risk of bias. In Additional file 1: Fig. S6, we show that our estimates are robust to excluding the locations with the fastest changing epidemics at the time of data collection, and are thus robust to the choice of dates for outcome data collection. Then, in Additional file 1: Fig. S7 we show that our results are robust to excluding the locations with the longest delays between epidemic wave and seroprevalence study, which are the most susceptible to seroreversion, and thus our estimates are not strongly affected by seroreversion. Finally, besides these comprehensive robustness analyses, a highlight of our study is the validation of our results with an independent estimation method, based on the ratio-of-ratios approach (Fig. 2).

Our results are highly relevant for aspects of COVID-19 modeling, such as estimating the number of unreported infections from hospital and ICU data, allowing to better estimate the present levels of natural immunity [4, 12, 13]; prediction of the effects of public-health policies implemented along the pandemic [14]; evaluating policy decisions such as vaccine allocation [15, 16]; the prediction of health outcomes in countries with high or low vaccination rates that account for the age-distribution of each country [17, 18]. Particularly, our estimates are important for analyzing the risk of COVID-19 for younger populations. These populations have very low risks of death, but as seen in our estimates, the risk of severe disease can be 2 orders of magnitude larger than the risk of death, and thus severe and critical outcomes are essential to properly characterize the risk of these populations. One illustrative example is the discussion around vaccination of young individuals against COVID-19. The FDA estimates that the rate of mRNA-vaccine-induced myocarditis, a side effect which is mild in some cases but which can result in severe outcomes, is of 1/5000 for males in ages between 16–17, the population at highest risk [19] (in line with reports from other locations such as Israel [20]). Although doing a risk–benefit analysis for adolescent vaccination is very complicated, and outside of the scope of this work, it is notable that while our estimated IFR is 0.12 [0.07–0.20] times the rate of vaccine induced myocarditis (i.e. 8 times smaller), our estimated ISR is 12 [7–19] times larger this rate. Thus, although such a direct comparison has many limitations, and should not be taken as a risk–benefit analysis, it shows how radically the conclusions of risk evaluation for young individuals depends on the disease outcome being considered.

Importantly, we note that the dynamic nature of the COVID-19 pandemic makes any estimates of outcome rates transient, since those rates are expected to change in space and time as new variants emerge and social behavior and medical practices change. As such, generalization of our estimates across time and space requires caution. For example, substantial drops in hospital and ICU mortality between the first and second waves have been reported in developed countries [21–26], partly due to improvements in care, although this is accompanied by considerable geographical heterogeneity [27]. On the other hand, the emergence of variants of concern was associated with increased rates of hospital mortality [28–30] and severe, critical and fatal cases [31–37] for early variants (Alpha, Gamma, Delta), and with a decrease in disease severity for the posterior variant Omicron [38]. Recent changes in disease severity are also due to the introduction of effective vaccines, with reductions in the rates of hospitalization and death of over 90% for the BNT162b2 vaccine [39]. More recently, oral antiviral medications have become available that can reduce the risk of hospitalization from COVID-19 close to 90% in patients at high risk of developing severe COVID-19 [40]. However, the effects of all of these changes on disease severity have been estimated in relative terms, rather than in absolute changes in risk. The estimates of severe and critical disease that we provide in this work can thus serve as the baseline to estimate the absolute risks of COVID-19 after such changes (e.g. the risk of severe disease for vaccinated individuals, or for unvaccinated individuals in the presence of Omicron). One particularly important problem where we may wish to calculate such absolute risks of COVID-19 severe and critical disease is to anticipate the effects of variants with potential for immune escape [41], or the effects of waning of vaccine effectiveness [42].

Finally, we note that the current work has some limitations that should be considered. One limitation is that our estimates rely on publicly available data on the number of hospitalizations, ICU admissions, and deaths, which may not match exactly the real number of severe and critical cases. Hospitalizations can underestimate the number of severe cases if a health system is overwhelmed, and some severe cases are not admitted or are not reported properly. On the other hand, the number of COVID-19-related hospitalizations may overestimate the number of severe cases if some people are admitted without severe COVID-19 infection, for example due to an abundance of caution in low-occupancy situations, or due to an incidental positive test at the time of admission in high-prevalence situations. Another limitation stems from the fact that the protocols for testing, admitting, and treating patients can vary between locations, and may depend on the strain of the healthcare system. For example, a large COVID-19 wave may induce a higher rate of severe disease, due to limited treatment capacity, and conversely to a smaller rate of hospital admissions due to limited resources. The underlying health of each population, and quality of care will also determine the outcome rates as specific locations. For example, race and socioeconomic status have been reported as risk factors for critical COVID-19 [43, 44], and striking differences in COVID-19 IFR between developed and developing countries have been reported [45]. In line with this, our statistical analysis shows that there is significant variability in the adverse outcome rates in between locations (in our models this is captured by the standard deviation of the intercept and slope of the log-ISR- and the log-ICR-age regression, see Additional file 1: Table S2). Thus, although we rigorously take variability between countries into account in our estimates, caution is required when extrapolating our estimates of the average ISR and ICR to specific locations.

## Supplementary Information

**Additional file 1**: Description of data: Robustness checks of the main analysis.

## Data Availability

All data and code used in this project are available at https://github.com/dherrera1911/estimate_covid_severity.

## Abbreviations

IFR: Infection fatality rate
ICR: Infection-critical rate
ISR: Infection-severe rate
FDA: Food and Drug Administration
ICU: Intensive Care Unit
SFR: Severe fatality rate

## References

[1] Levin AT, Hanage WP, Owusu-Boaitey N, Cochran KB, Walsh SP, Meyerowitz-Katz G (2020). Assessing the age specificity of infection fatality rates for COVID-19: systematic review, meta-analysis, and public policy implications. Eur J Epidemiol.

[2] O’Driscoll M, Dos Santos GR, Wang L, Cummings DAT, Azman AS, Paireau J, et al. Age-specific mortality and immunity patterns of SARS-CoV-2. Nature [Internet]. 2020 Nov 2 [cited 2020 Nov 5]; Available from: http://www.nature.com/articles/s41586-020-2918-0. 10.1038/s41586-020-2918-0 33137809

[3] Brazeau N, Verity R, Jenks S, Fu H, Whittaker C, Winskill P, et al. Report 34: COVID-19 infection fatality ratio: estimates from seroprevalence [Internet]. Imperial College London; 2020 Oct [cited 2020 Nov 2]. Available from: http://spiral.imperial.ac.uk/handle/10044/1/83545.

[4] Hozé N, Paireau J, Lapidus N, Tran Kiem C, Salje H, Severi G (2021). Monitoring the proportion of the population infected by SARS-CoV-2 using age-stratified hospitalisation and serological data: a modelling study. Lancet Public Health.

[5] Lapidus N, Paireau J, Levy-Bruhl D, de Lamballerie X, Severi G, Touvier M (2021). Do not neglect SARS-CoV-2 hospitalization and fatality risks in the middle-aged adult population. Infect Dis Now.

[6] Espenhain L, Tribler S, Sværke Jørgensen C, Holm Hansen C, Wolff Sönksen U, Ethelberg S (2021). Prevalence of SARS-CoV-2 antibodies in Denmark: nationwide, population-based seroepidemiological study. Eur J Epidemiol.

[7] Menachemi N, Dixon BE, Wools-Kaloustian KK, Yiannoutsos CT, Halverson PK (2021). How many SARS-CoV-2-infected people require hospitalization? Using random sample testing to better inform preparedness efforts. J Public Health Manag Pract.

[8] Mahajan S, Caraballo C, Li S-X, Dong Y, Chen L, Huston SK (2021). SARS-CoV-2 infection hospitalization rate and infection fatality rate among the non-congregate population in Connecticut. Am J Med.

[9] Seedat S, Chemaitelly H, Ayoub HH, Makhoul M, Mumtaz GR, Al Kanaani Z (2021). SARS-CoV-2 infection hospitalization, severity, criticality, and fatality rates in Qatar. Sci Rep.

[10] Verity R, Okell LC, Dorigatti I, Winskill P, Whittaker C, Imai N, et al. Estimates of the severity of coronavirus disease 2019: a model-based analysis. Lancet Infect Dis [Internet]. 2020 Mar 30 [cited 2020 Apr 3]; Available from: http://www.sciencedirect.com/science/article/pii/S1473309920302437. 10.1016/S1473-3099(20)30243-7 PMC7158570 32240634

[11] Blackburn J, Yiannoutsos CT, Carroll AE, Halverson PK, Menachemi N (2021). Infection fatality ratios for COVID-19 among noninstitutionalized persons 12 and older: results of a random-sample prevalence study. Ann Intern Med.

[12] Irons NJ, Raftery AE. Estimating SARS-CoV-2 infections from deaths, confirmed cases, tests, and random surveys. Proc Natl Acad Sci [Internet]. 2021 Aug 3 [cited 2021 Jul 28];118(31). Available from: https://www.pnas.org/content/118/31/e2103272118. 10.1073/pnas.2103272118 PMC8346866 34312227

[13] Russell TW, Golding N, Hellewell J, Abbott S, Wright L, Pearson CAB (2020). Reconstructing the early global dynamics of under-ascertained COVID-19 cases and infections. BMC Med.

[14] Davies NG, Barnard RC, Jarvis CI, Russell TW, Semple MG, Jit M (2021). Association of tiered restrictions and a second lockdown with COVID-19 deaths and hospital admissions in England: a modelling study. Lancet Infect Dis.

[15] Hjorleifsson KE, Rognvaldsson S, Jonsson H, Agustsdottir AB, Andresdottir M, Birgisdottir K, et al. Reconstruction of a large-scale outbreak of SARS-CoV-2 infection in Iceland informs vaccination strategies. Clin Microbiol Infect [Internet]. 2022 Feb 16 [cited 2022 Feb 25];0(0). Available from: https://www.clinicalmicrobiologyandinfection.com/article/S1198-743X(22)00085-4/fulltext. 10.1016/j.cmi.2022.02.012 PMC8849849 35182757

[16] Matrajt L, Eaton J, Leung T, Brown ER (2020). Vaccine optimization for COVID-19: who to vaccinate first?. Sci Adv.

[17] Davies NG, Kucharski AJ, Eggo RM, Gimma A, Edmunds WJ, Jombart T, et al. Effects of non-pharmaceutical interventions on COVID-19 cases, deaths, and demand for hospital services in the UK: a modelling study. Lancet Public Health [Internet]. 2020 Jun 2 [cited 2020 Jun 25]; Available from: http://www.sciencedirect.com/science/article/pii/S246826672030133X. 10.1016/S2468-2667(20)30133-X PMC7266572 32502389

[18] Sandmann FG, Davies NG, Vassall A, Edmunds WJ, Jit M, Sun FY (2021). The potential health and economic value of SARS-CoV-2 vaccination alongside physical distancing in the UK: a transmission model-based future scenario analysis and economic evaluation. Lancet Infect Dis.

[19] FDA. August 23, 2021 Summary basis for regulatory action—comirnaty [Internet]. [cited 2021 Sep 30]. Available from: https://www.fda.gov/media/151733/.

[20] Mevorach D, Anis E, Cedar N, Bromberg M, Haas EJ, Nadir E (2021). Myocarditis after BNT162b2 mRNA vaccine against Covid-19 in Israel. N Engl J Med.

[21] Anesi GL, Jablonski J, Harhay MO, Atkins JH, Bajaj J, Baston C (2021). Characteristics, outcomes, and trends of patients with COVID-19-related critical illness at a learning health system in the United States. Ann Intern Med.

[22] Asch DA, Sheils NE, Islam MN, Chen Y, Werner RM, Buresh J (2021). Variation in US hospital mortality rates for patients admitted with COVID-19 during the first 6 months of the pandemic. JAMA Intern Med.

[23] Aznar-Gimeno R, Paño-Pardo JR, Esteban LM, Labata-Lezaun G, Esquillor-Rodrigo MJ, Lanas A (2021). Changes in severity, mortality, and virus genome among a Spanish cohort of patients hospitalized with SARS-CoV-2. Sci Rep.

[24] Navaratnam AV, Gray WK, Day J, Wendon J, Briggs TWR (2021). Patient factors and temporal trends associated with COVID-19 in-hospital mortality in England: an observational study using administrative data. Lancet Respir Med.

[25] Doidge JC, Gould DW, Ferrando-Vivas P, Mouncey PR, Thomas K, Shankar-Hari M (2021). Trends in intensive care for patients with COVID-19 in England, Wales, and Northern Ireland. Am J Respir Crit Care Med.

[26] Armstrong RA, Kane AD, Kursumovic E, Oglesby FC, Cook TM (2021). Mortality in patients admitted to intensive care with COVID-19: an updated systematic review and meta-analysis of observational studies. Anaesthesia.

[27] Kadri SS, Sun J, Lawandi A, Strich JR, Busch LM, Keller M (2021). Association between caseload surge and COVID-19 survival in 558 U.S. Hospitals, March to August 2020. Ann Intern Med.

[28] Freitas ARR, Beckedorff OA, Cavalcanti de LPG, Siqueira AM, Castro de DB, Costa da CF (2021). The emergence of novel SARS-CoV-2 variant P.1 in Amazonas (Brazil) was temporally associated with a change in the age and sex profile of COVID-19 mortality: a population based ecological study. Lancet Reg Health Am..

[29] Jassat W, Mudara C, Ozougwu L, Tempia S, Blumberg L, Davies M-A, et al. Increased mortality among individuals hospitalised with COVID-19 during the second wave in South Africa 2021. 10.1101/2021.03.09.21253184v1.

[30] Khedar RS, Mittal K, Ambaliya HC, Mathur A, Gupta JB, Sharma KK, et al. Greater COVID-19 severity and mortality in hospitalized patients in second (delta variant) wave compared to the first: single centre prospective study in India. 2021. 10.1101/2021.09.03.21263091v1.

[31] Challen R, Brooks-Pollock E, Read JM, Dyson L, Tsaneva-Atanasova K, Danon L (2021). Risk of mortality in patients infected with SARS-CoV-2 variant of concern 202012/1: matched cohort study. BMJ.

[32] Davies NG, Jarvis CI, Edmunds WJ, Jewell NP, Diaz-Ordaz K, Keogh RH (2021). Increased mortality in community-tested cases of SARS-CoV-2 lineage B.1.1.7. Nature.

[33] Fisman DN, Tuite AR. Progressive increase in virulence of novel SARS-CoV-2 variants in Ontario, Canada 2021. 10.1101/2021.07.05.21260050v3.

[34] Fisman DN, Tuite AR. Age-specific changes in virulence associated with SARS-CoV-2 variants of concern. 2021; 10.1101/2021.09.25.21264097v1. 10.1093/cid/ciac174 PMC9047153 35234859

[35] Grint DJ, Wing K, Williamson E, McDonald HI, Bhaskaran K, Evans D (2021). Case fatality risk of the SARS-CoV-2 variant of concern B.1.1.7 in England, 16 November to 5 February. Eurosurveillance.

[36] Ong SWX, Chiew CJ, Ang LW, Mak T-M, Cui L, Toh MPH, et al. Clinical and virological features of SARS-CoV-2 variants of concern: a retrospective cohort study comparing B.1.1.7 (Alpha), B.1.315 (Beta), and B.1.617.2 (Delta) [Internet]. Rochester, NY: Social Science Research Network; 2021 Jun [cited 2021 Sep 30]. Report No.: ID 3861566. Available from: https://papers.ssrn.com/abstract=3861566.

[37] Tuite AR, Fisman DN, Odutayo A, Bobos P, Allen V, Bogoch II, et al. COVID-19 Hospitalizations, ICU admissions and deaths associated with the new variants of concern [Internet]. Ontario COVID-19 Science Advisory Table; 2021 Mar [cited 2021 Sep 30]. Available from: https://covid19-sciencetable.ca/sciencebrief/covid-19-hospitalizations-icu-admissions-and-deaths-associated-with-the-new-variants-of-concern.

[38] Iuliano AD. Trends in disease severity and health care utilization during the early omicron variant period compared with previous SARS-CoV-2 High Transmission Periods—United States, December 2020–January 2022. MMWR Morb Mortal Wkly Rep [Internet]. 2022 [cited 2022 Feb 23];71. Available from: https://www.cdc.gov/mmwr/volumes/71/wr/mm7104e4.htm. 10.15585/mmwr.mm7104e4 PMC9351529 35085225

[39] Andrews N, Tessier E, Stowe J, Gower C, Kirsebom F, Simmons R (2022). Duration of protection against mild and severe disease by COVID-19 vaccines. N Engl J Med.

[40] Hammond J, Leister-Tebbe H, Gardner A, Abreu P, Bao W, Wisemandle W, et al. Oral nirmatrelvir for high-risk, nonhospitalized adults with COVID-19. N Engl J Med. 2022. 10.1056/NEJMoa2118542 PMC8908851 35172054

[41] Collie S, Champion J, Moultrie H, Bekker L-G, Gray G (2022). Effectiveness of BNT162b2 vaccine against omicron variant in South Africa. N Engl J Med.

[42] Feikin DR, Higdon MM, Abu-Raddad LJ, Andrews N, Araos R, Goldberg Y, et al. Duration of effectiveness of vaccines against SARS-CoV-2 infection and COVID-19 disease: results of a systematic review and meta-regression. The Lancet [Internet]. 2022 Feb 21 [cited 2022 Feb 23];0(0). Available from: https://www.thelancet.com/journals/lancet/article/PIIS0140-6736(22)00152-0/fulltext. 10.1016/S0140-6736(22)00152-0 PMC8863502 35202601

[43] Quan D, Luna Wong L, Shallal A, Madan R, Hamdan A, Ahdi H (2021). Impact of race and socioeconomic status on outcomes in patients hospitalized with COVID-19. J Gen Intern Med.

[44] Patel AP, Paranjpe MD, Kathiresan NP, Rivas MA, Khera AV (2020). Race, socioeconomic deprivation, and hospitalization for COVID-19 in English participants of a national biobank. Int J Equity Health.

[45] Levin AT, Owusu-Boaitey N, Pugh S, Fosdick BK, Zwi AB, Malani A (2021). Assessing the burden of COVID-19 in developing countries: systematic review, meta-analysis, and public policy implications. medRxiv..

[46] Gudbjartsson DF, Norddahl GL, Melsted P, Gunnarsdottir K, Holm H, Eythorsson E (2020). Humoral immune response to SARS-CoV-2 in Iceland. N Engl J Med.

[47] Waterfield T, Watson C, Moore R, Ferris K, Tonry C, Watt A (2021). Seroprevalence of SARS-CoV-2 antibodies in children: a prospective multicentre cohort study. Arch Dis Child.

[48] Ward H, Atchison C, Whitaker M, Ainslie KEC, Elliott J, Okell L (2021). SARS-CoV-2 antibody prevalence in England following the first peak of the pandemic. Nat Commun.

[49] Carrat F, de Lamballerie X, Rahib D, Blanché H, Lapidus N, Artaud F, et al. Seroprevalence of SARS-CoV-2 Among Adults in Three Regions of France Following the Lockdown and Associated Risk Factors: A Multicohort Study [Internet]. Rochester, NY: Social Science Research Network; 2020 Oct [cited 2021 Jan 6]. Report No.: ID 3696820. Available from: https://papers.ssrn.com/abstract=3696820.

[50] Ireland Health Service Executive. Preliminary report of the results of the Study to Investigate COVID-19 Infection in People Living in Ireland (SCOPI): a national seroprevalence study, June-July 2020 [Internet]. 2020 [cited 2021 Jul 4]. Available from: https://www.hpsc.ie/a-z/respiratory/coronavirus/novelcoronavirus/scopi/.

[51] Vos ERA, den Hartog G, Schepp RM, Kaaijk P, van Vliet J, Helm K (2020). Nationwide seroprevalence of SARS-CoV-2 and identification of risk factors in the general population of the Netherlands during the first epidemic wave. J Epidemiol Commun Health..

[52] Pollán M, Pérez-Gómez B, Pastor-Barriuso R, Oteo J, Hernán MA, Pérez-Olmeda M (2020). Prevalence of SARS-CoV-2 in Spain (ENE-COVID): a nationwide, population-based seroepidemiological study. The Lancet.

[53] Biggs HM. Estimated community seroprevalence of SARS-CoV-2 antibodies—two Georgia Counties, April 28–May 3, 2020. MMWR Morb Mortal Wkly Rep [Internet]. 2020 [cited 2021 Jan 20];69. Available from: https://www.cdc.gov/mmwr/volumes/69/wr/mm6929e2.htm. 10.15585/mmwr.mm6929e2 PMC7377817 32701941

[54] Rosenberg ES, Tesoriero JM, Rosenthal EM, Chung R, Barranco MA, Styer LM (2020). Cumulative incidence and diagnosis of SARS-CoV-2 infection in New York. Ann Epidemiol.

[55] Ontario Public Health. COVID-19 Seroprevalence in Ontario: July 4 to July 31, 2020 [Internet]. 2020 p. 9. Available from: https://www.publichealthontario.ca/-/media/documents/ncov/epi/2020/10/covid-19-epi-seroprevalence-in-ontario-july-31.pdf?la=en.

[56] Sweden Public Health Authority. Veckorapport om covid-19, vecka 24 [Internet]. 2020 [cited 2021 Jul 4]. Available from: https://www.folkhalsomyndigheten.se/globalassets/statistik-uppfoljning/smittsamma-sjukdomar/veckorapporter-covid-19/2020/covid-19-veckorapport-vecka-24_final.pdf.

[57] Perez-Saez J, Lauer SA, Kaiser L, Regard S, Delaporte E, Guessous I, et al. Serology-informed estimates of SARS-CoV-2 infection fatality risk in Geneva, Switzerland. Lancet Infect Dis [Internet]. 2020 Jul 14 [cited 2021 Jan 17];0(0). Available from: https://www.thelancet.com/journals/laninf/article/PIIS1473-3099(20)30584-3/abstract. 10.1016/S1473-3099(20)30584-3 PMC7833057 32679085

[58] Herzog S, Bie JD, Abrams S, Wouters I, Ekinci E, Patteet L (2020). Seroprevalence of IgG antibodies against SARS coronavirus 2 in Belgium—a serial prospective cross-sectional nationwide study of residual samples. medRxiv..

[59] Mahajan S, Srinivasan R, Redlich CA, Huston SK, Anastasio KM, Cashman L (2021). Seroprevalence of SARS-CoV-2-Specific IgG antibodies among adults living in Connecticut: post-infection prevalence (PIP) study. Am J Med.

[60] Chishinga N, Gandhi NR, Onwubiko UN, Telford C, Prieto J, Smith S, et al. Characteristics and risk factors for hospitalization and mortality among persons with COVID-19 in Atlanta Metropolitan Area. Infect Diseases (except HIV/AIDS). 2020. 10.1101/2020.12.15.20248214.

[61] Molenberghs G, Faes C, Aerts J, Theeten H, Devleesschauwer B, Sierra NB (2020). Belgian COVID-19 mortality, excess deaths, number of deaths per million, and infection fatality rates (8 March–9 May 2020). medRxiv.

[62] Eythorsson E, Helgason D, Ingvarsson RF, Bjornsson HK, Olafsdottir LB, Bjarnadottir V (2020). Clinical spectrum of coronavirus disease 2019 in Iceland: population based cohort study. BMJ.

[63] Park H-Y, Lee JH, Lim N-K, Lim DS, Hong SO, Park M-J, et al. Presenting characteristics and clinical outcome of patients with COVID-19 in South Korea: A nationwide retrospective observational study. Lancet Reg Health—West Pac [Internet]. 2020 Dec 1 [cited 2021 Jan 5];5. Available from: https://www.thelancet.com/journals/lanwpc/article/PIIS2666-6065(20)30061-4/abstract. 10.1016/j.lanwpc.2020.100061 PMC7691821 34173605

[64] Carpenter B, Gelman A, Hoffman MD, Lee D, Goodrich B, Betancourt M (2017). Stan: a probabilistic programming language. J Stat Softw.

[65] Cummings MJ, Baldwin MR, Abrams D, Jacobson SD, Meyer BJ, Balough EM (2020). Epidemiology, clinical course, and outcomes of critically ill adults with COVID-19 in New York City: a prospective cohort study. The Lancet.

[66] COVID-ICU Group on behalf of the REVA Network and the COVID-ICU Investigators. Clinical characteristics and day-90 outcomes of 4244 critically ill adults with COVID-19: a prospective cohort study. Intensive Care Med. 2021;47(1):60–73. 10.1007/s00134-020-06294-x PMC7674575 33211135

[67] Ranzani OT, Bastos LSL, Gelli JGM, Marchesi JF, Baião F, Hamacher S (2021). Characterisation of the first 250 000 hospital admissions for COVID-19 in Brazil: a retrospective analysis of nationwide data. Lancet Respir Med.

[68] Oliveira E, Parikh A, Lopez-Ruiz A, Carrilo M, Goldberg J, Cearras M (2021). ICU outcomes and survival in patients with severe COVID-19 in the largest health care system in central Florida. PLoS ONE.

[69] González-Dambrauskas S, Vásquez-Hoyos P, Camporesi A, Díaz-Rubio F, Piñeres-Olave BE, Fernández-Sarmiento J (2020). Pediatric critical care and COVID-19. Pediatrics.

[70] Prata-Barbosa A, Lima-Setta F, dos Santos GR, Lanziotti VS, de Castro REV, de Souza DC (2020). Pediatric patients with COVID-19 admitted to intensive care units in Brazil: a prospective multicenter study. J Pediatr (Rio J).

[71] Richardson S, Hirsch JS, Narasimhan M, Crawford JM, McGinn T, Davidson KW (2020). Presenting characteristics, comorbidities, and outcomes among 5700 patients hospitalized with COVID-19 in the New York City area. JAMA.

[72] Karagiannidis C, Mostert C, Hentschker C, Voshaar T, Malzahn J, Schillinger G (2020). Case characteristics, resource use, and outcomes of 10 021 patients with COVID-19 admitted to 920 German hospitals: an observational study. Lancet Respir Med.

[73] Salje H, Kiem CT, Lefrancq N, Courtejoie N, Bosetti P, Paireau J, et al. Estimating the burden of SARS-CoV-2 in France. Science [Internet]. 2020 May 13 [cited 2020 May 13]; Available from: https://science.sciencemag.org/content/early/2020/05/12/science.abc3517. 10.1126/science.abc3517 PMC7223792 32404476

[74] Docherty AB, Harrison EM, Green CA, Hardwick HE, Pius R, Norman L (2020). Features of 20 133 UK patients in hospital with COVID-19 using the ISARIC WHO clinical characterisation protocol: prospective observational cohort study. BMJ.

[75] Berenguer J, Ryan P, Rodríguez-Baño J, Jarrín I, Carratalà J, Pachón J (2020). Characteristics and predictors of death among 4035 consecutively hospitalized patients with COVID-19 in Spain. Clin Microbiol Infect.

[76] Maquilon C, Gongora J, Antolini M, Alvarado B, Valdes N, Benavente A, et al. Risk factors on admission and condition at discharge of 529 consecutive COVID-19 patients at a tertiary care center in Santiago, Chile [Internet]. In Review; 2020 Sep [cited 2021 Jun 15]. Available from: https://www.researchsquare.com/article/rs-71187/v1.

